# Knockdown of Placental Major Facilitator Superfamily Domain Containing 2a in Pregnant Mice Reduces Fetal Brain Growth and Phospholipid Docosahexaenoic Acid Content

**DOI:** 10.3390/nu15234956

**Published:** 2023-11-29

**Authors:** Theresa L. Powell, Kenneth Barentsen, Owen Vaughan, Charis Uhlson, Karin Zemski Berry, Kathryn Erickson, Kelsey Faer, Stephanie S. Chassen, Thomas Jansson

**Affiliations:** 1 Department of Pediatrics, University of Colorado Anschutz Medical Campus, 13001 E 17th Pl, Aurora, CO 80045, USA; 2 Department of Obstetrics and Gynecology, University of Colorado Anschutz Medical Campus, 13001 E 17th Pl, Aurora, CO 80045, USA; 3 Department of Maternal and Fetal Medicine, EGA Institute for Women’s Heath, University College London, 86-96 Chenies Mews, London WC1E 6HX, UK; 4 Department of Medicine, University of Colorado Anschutz Medical Campus, 13001 E 17th Pl, Aurora, CO 80045, USA

**Keywords:** maternal–fetal exchange, fatty acids, docosahexaenoic acids, trophoblast, pregnancy

## Abstract

Introduction: Docosahexaenoic acid (DHA) is an *n*-3 long chain polyunsaturated fatty acid critical for fetal brain development that is transported to the fetus from the mother by the placenta. The lysophosphatidylcholine (LPC) transporter, Major Facilitator Superfamily Domain Containing 2a (MFSD2a), is localized in the basal plasma membrane of the syncytiotrophoblast of the human placenta, and MFSD2a expression correlates with umbilical cord blood LPC-DHA levels in human pregnancy. We hypothesized that placenta-specific knockdown of MFSD2a in pregnant mice reduces phospholipid DHA accumulation in the fetal brain. Methods: Mouse blastocysts (E3.5) were transduced with an EGFP-expressing lentivirus containing either an shRNA targeting MFSD2a or a non-coding sequence (SCR), then transferred to pseudopregnant females. At E18.5, fetuses were weighed and their placenta, brain, liver and plasma were collected. MFSD2a mRNA expression was determined by qPCR in the brain, liver and placenta and phospholipid DHA was quantified by LC-MS/MS. Results: MFSD2a-targeting shRNA reduced placental mRNA MFSD2a expression by 38% at E18.5 (*n* = 45, *p* < 0.008) compared with SCR controls. MFSD2a expression in the fetal brain and liver were unchanged. Fetal brain weight was reduced by 13% (*p* = 0.006). Body weight, placenta and liver weights were unaffected. Fetal brain phosphatidyl choline and phosphatidyl ethanolamine DHA content was lower in fetuses with placenta-specific MFSD2a knockdown. Conclusions: Placenta-specific reduction in expression of the LPC-DHA transporter MFSD2a resulted in reduced fetal brain weight and lower phospholipid DHA content in the fetal brain. These data provide mechanistic evidence that placental MFSD2a mediates maternal–fetal transfer of LPC-DHA, which is critical for brain growth.

## 1. Introduction

The critical need for long chain polyunsaturated fatty acids (LCPUFAs) during pregnancy is well established [1,2]. Fetal and placental de novo synthesis of LCPUFAs, such as synthesis of docosahexaenoic acid (DHA, 22:6, *n*-3) from the essential fatty acid alpha linoleic acid (18:3, *n*-3), is inadequate to sustain normal growth and development of the fetal brain and retina [3]. The brain is second only to adipose tissue in lipid content [4], and the level of DHA is high in neuronal tissues such as the retina and cerebral cortex and at synapses [5,6]. Therefore, transfer of these lipids from the maternal circulation across the placenta is essential both for in utero brain development and long-term neurological health of the individual. In animal models, maternal dietary DHA deficiencies are detrimental to fetal brain growth and development, neurogenesis, cognitive function and vision [7,8,9]. Importantly, perinatal DHA status has also been reported to be associated with neurological function in children [10,11].

Dietary supplementation with DHA in pregnancy has been recommended by the American Academy of Pediatrics, the American College of Obstetricians and Gynecologists, and March of Dimes [12]. The accumulation of DHA in the developing fetal brain is greatest in the third trimester when approximately 50 mg are deposited daily [10]. Low maternal DHA status in human pregnancy is associated with learning, cognitive, motor and visual disorders in children (for a review, see [10]). Evidence that DHA supplementation in pregnancy improves cognitive function, learning or vision in the children of supplemented women has been inconsistent, with some studies showing improvement but not all [13,14,15]. A study of women with Gestational Diabetes Mellitus (GDM) found that the mothers had higher DHA dietary intake and RBC levels, while their infants were deficient in DHA and suffered from increased neurological sequelae [16]. These studies suggest that placental handling and transfer of DHA plays a critical role in determining fetal DHA availability.

In 2014, Nguyen and coworkers reported that Major Facilitator Superfamily domain-containing 2a (MFSD2a) is expressed in the endothelial cells of the blood–brain barrier and provided evidence that it transports DHA in the form of lysophosphatidylcholine (LPC-DHA) [17]. Mice deficient in MFSD2a have severe microcephaly, brain DHA deficiency, and learning and memory deficits [18]. The role of placental MFSD2a in the delivery of DHA to the fetal brain is currently unknown. We hypothesized that placenta-specific knockdown of MFSD2a in pregnant mice reduces phospholipid DHA accumulation in the fetal brain. We used a mouse model of blastocyst trophectoderm lentivirus transduction to achieve placenta-specific knockdown of MFSD2a and measured brain growth and phospholipid DHA content at the end of gestation. 

## 2. Materials and Methods 

### 2.1. Lentiviral Vectors

The custom shRNA lentivirus for gene silencing, targeting mouse Mfsd2a mRNA (Mfsd2aKD) and based upon pLKO.1, the lentivirus bicistronic transgene, pLV[shRNA]-EGFP-U6>mMfsd2a[shRNA#3] was obtained from a commercial vendor (VectorBuilder Inc., Chicago, IL, USA). The targeting sequence (GCTTACTTCCTCATCTGGTTT) was located downstream of the human U6 small nuclear 1 promoter. The plasmid backbone also contained an enhanced green fluorescent protein (EGFP) open reading frame expressed from the human phosphoglycerate kinase 1 promoter and an ampicillin resistance gene. A control pLV[shRNA]-EGFP-U6>Scramble_shRNA lentivirus (SCR), with a non-coding targeting sequence (CCTAAGGTTAAGTCGCCCTCG), was also obtained from a commercial vendor (VectorBuilder Inc., Chicago, IL, USA). The lentiviral vectors were both titrated by transducing 293FT cells with serial dilutions of the viral suspension then determining the percentage of EGFP fluorescent cells using flow cytometry.

### 2.2. Animals

All procedures were approved by the Institutional Animal Care and Use Committee of the University of Colorado (protocol # 344) and described for knocking down placental SNAT2 amino acid transporter in our previous publication [19]. The animals were purchased from Charles River (Wilmington, MA, USA) or Jackson Laboratories (Bar Harbor, ME, USA) and maintained under standard 14:10 h light: dark conditions with ad libitum access to food and water. Female B6D2F1 (strain code 100006) mice aged <3 weeks were injected with pregnant mare serum gonadotrophin (5 I.U., i.p., Prospec, East Brunswick, NJ, USA) and human chorionic gonadotrophin (5 I.U., i.p., Sigma-Aldrich, St. Louis, MO, USA) to induce superovulation and subsequently mated overnight with stud B6D2F1 males aged 8–56 weeks. Successfully mated females were identified by the presence of a copulatory plug the following morning, and this was designated as embryonic day (E) 0.5 (term ≈ E19.5). On E3.5, pregnant females were euthanized by CO_2_ asphyxiation and cervical dislocation, their uteri removed, and each horn flushed with 0.5 mL of pre-warmed M2 medium (M7167, Sigma-Aldrich, St. Louis, MO, USA). The zona pellucida was removed by serial submersion in three drops of acidic Tyrodes solution (~10 s each). The embryos were then thoroughly washed and incubated in batches in embryo culture medium (EmbryoMax Advanced KSOM Embryo medium, MR-101-D, Millipore, Burlington, MA, USA). With the trophectoderm exposed, the blastocysts were transduced with either Mfsd2aKD or SCR by incubating batches of 5–10 blastocysts with 5 × 10^6^ transforming units of lentivirus diluted in KSOM in a total volume of 20 µL for 4 h. The blastocysts were then removed from the transducing drops and washed in 12 drops of KSOM and surgically transferred to 14 pseudopregnant female CD-1 IGS recipients (strain code 022). All in vitro manipulations were performed at 37 °C and under low light conditions. The female CD-1 embryo recipient mice were identified by copulatory plug after mating with vasectomized B6D2F1 males overnight to induce pseudopregnancy. The pseudopregnant females underwent surgical embryo transfer 2.5 days post-copulatory plug. The recipients were given pre-operative analgesia (meloxicam, 1 mg/kg, i.p.), then anaesthetized with isofluorane (2%, inhaled). With the animal in sternal recumbency and under aseptic conditions, a 0.5 cm incision was made in the skin of the flank overlying the right ovary, 1 cm caudal to the rib cage. The ovary, oviduct and distal uterine horn were exteriorized through the body wall and a 26-gauge needle was used to puncture the uterus below the oviductal junction. Ten lentivirus transduced blastocysts were aspirated into the uterine horn in a minimal volume of culture medium. The uterus and oviducts were reintegrated into the body cavity, the muscle wall closed with a single absorbable suture (5.0 vicryl) and the skin fastened with a 9 mm wound clip. The procedure was then repeated for the left ovary, with Mfsd2a and SCR transduced blastocysts transferred to contralateral horns and randomized to the left and right in each recipient. Post-operatively, the recipient females were recovered from anesthesia on a heated mat and housed as pairs in static caging. At E18.5, the dams were euthanized for collection of fetal and placental tissues. Following laparotomy, the fetuses and placentas were removed and weighed. The placentas from each uterine horn were pooled and observed under a fluorescent microscope to visualize placenta-specific GFP reporter expression and remove any non-transduced fetal/placental dyads. The fetal tissues were dissected, frozen in liquid nitrogen and stored at −80 °C until batch analysis could be performed. Each uterine horn was examined for implantation sites to determine the implantation success rates for each horn based on the number of embryos transferred.

### 2.3. Gene Expression Analysis

RNA was extracted from the frozen placentas and fetal tissues, and reverse transcribed using commercially available kits (RNeasy Plus Mini kit, Qiagen and High-Capacity cDNA RT kit, Invitrogen). The expression of *Mfsd2a* was determined by SYBR green qRT-PCR using the relative standard curve method, relative to *Rpl19* RNA. The primer sequences are given in Supplementary Table S1.

### 2.4. Placental and Brain Lipid Extraction and Phospholipid Analysis

Lipids were extracted from the mouse brains and placentas (15 mg) with a combination of water (750 μL), methanol (900 μL) and methyl-*tert*-butyl ether (3 mL) [20] after the addition of an internal standard cocktail containing phosphatidyl choline (PC) 19:0/19:0 (2000 pmol), d7-PC-18:1/OH (200 pmol), PE-17:0/17:0 (1000 pmol), and phosphatidyl ethanolamine (PE)-17:1/OH (100 pmol). For phospholipid analysis, the samples were injected into an HPLC system connected to a triple quadrupole mass spectrometer (Sciex 4000 QTRAP, Framingham, MA, USA) and normal phase chromatography was performed using a silica column (150 × 2 mm, Luna Silica 5 µm, Phenomenex, Torrance, CA, USA). The mobile phase system consisted of solvent A (isopropanol/hexane/water (58/40/2, *v*/*v*)) and 35% solvent B (hexane/isopropanol/water (300/400/84, *v*/*v*/*v*)), both containing 10 mM ammonium acetate. Mass spectrometric analysis was performed for 67 molecular species of PE and PC diacyl and ether-containing lipids in the negative ion mode using multiple reaction monitoring (MRM). This method monitors a set of collision-induced mass transitions corresponding to the production of 16:0 (*m/z* 255.2), 16:1 (*m/z* 253.2), 18:0 (*m/z* 283.2), 18:1 (*m/z* 281.2), 18:2 (*m/z* 279.2), 20:4 (*m/z* 303.2), 20:5 (*m/z* 301.2), 22:4 (*m/z* 331.2), 22:5 (*m/z* 329.2), and 22:6 (*m/z* 327.2) carboxylate anions from the [M–H]^−^ of PE and lyso phosphatidyl ethanolamine (LPE) and the acetate adducts [M + CH_3_COO]^−^ for PC and lyso phosphatidyl choline (LPC) molecular species. The quantitative results were determined using stable isotope dilution with standard curves for saturated and unsaturated PC and PE molecular species.

### 2.5. Statistics 

We recovered fetuses from a total of 12 dams: 45 were MFSD2a knockdown and 34 were non-coding scramble (SCR) controls. The fetuses were batch-transduced in vitro and transferred into pseudopregnant recipients. The fetuses were treated as independent due to the variability in MFSD2a expression after lentivirus transduction. Two-tailed t-tests were used to test for differences in phospholipid levels between MFSD2a knockdown fetuses and scramble controls. Pearson’s correlation was used to test for correlation between MFSD2a RNA expression in the placenta and fetal brain phospholipid DHA content.

## 3. Results

**Figure 1:**
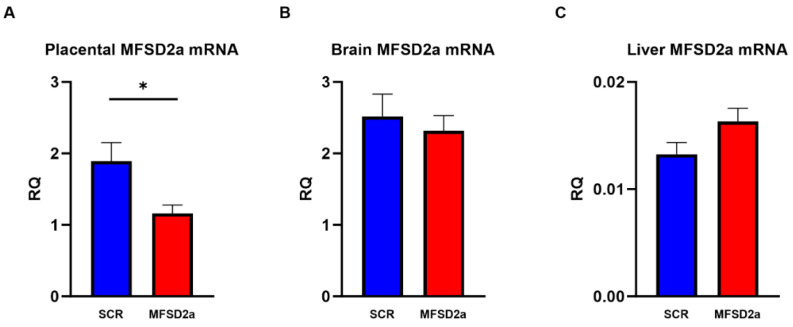
Relative Quantity (RQ) of mRNA for MFSD2a compared with the housekeeping gene RP1 mRNA measured by real-time PCR in (**A**) placenta, (**B**) fetal brain and (**C**) liver at E18.5 in fetuses with placental shRNA-induced MFSD2a knockdown (*n* = 45) and SCR non-coding controls (*n* = 34). Two sample unpaired *t*-tests, * = *p* < 0.05.

Fourteen dams were implanted with 20 blastocysts, 10 in each horn, for a total of 280 implanted embryos. Twelve dams remained pregnant to E18.5, an 86% success rate. Implantation rates after blastocyst lentivirus transduction and embryo transfer were within the normal range for experimental embryo transfer (*n* = 14 dams, 44 of 140 SCR and 53 of 140 MFSD2a transduced, 36% implantation rate overall). From the implanted blastocysts we collected 37 SCR pups and 48 MFSD2a pups at E18.5, which corresponds to 35% of transfected embryos surviving to term gestation. At E18.5, all the fetal/placental pairs were examined for EGFP expression, and we found that only the placenta expressed EGFP (Supplemental Figure S1). Those without EGFP placental staining were not used in the analysis. Placental *Mfsd*2a mRNA expression was reduced by 38% (*p* < 0.05) in the MFSD2a shRNA transfection (*n* = 45) group compared with the SCR controls (*n* = 34, Figure 1A). Fetal brain *Mfsd*2a mRNA expression was not different between the two groups (Figure 1B), nor was *Mfsd*2a mRNA expression different in the fetal liver (Figure 1C). These data are consistent with a placenta-specific reduction in *Mfsd*2a expression in our model and normal gene expression in the fetus.

**Figure 2:**
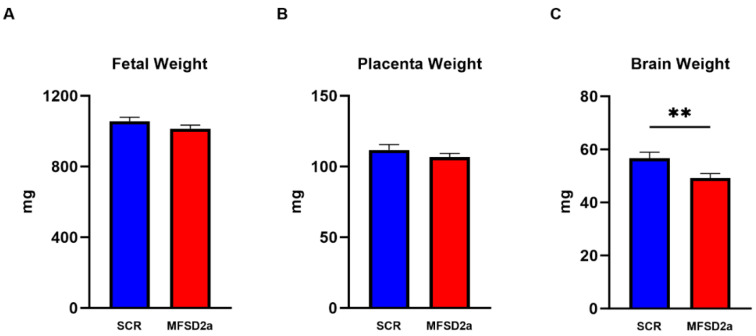
Fetal (**A**) body weight, (**B**) placenta weight and (**C**) brain weight at E18.5. Two sample unpaired *t*-Test, ** = *p* < 0.01.

We terminated the experiments at E18.5 and collected fetuses and placentas from each horn. There were no significant differences in fetal weight or placenta weight (Figure 2A,B) between the groups with MFSD2a placental knockdown (n = 45) and the SCR controls (*n* = 34). However, we found a 13% (*p* < 0.01) reduction in brain weight (Figure 2C) in the fetuses with placenta-specific MFSD2a knockdown. In order to evaluate weight changes in other fetal organs that were not impacted by changes in MFSD2a expression, we examined fetal liver weights, which were not different between groups (48.6 ± 2.4 mg, *n* = 36 SCR vs. 43.25 + 2.2 mg *n* = 48 MFSD2a KD, *p* = 0.11).

**Figure 3:**
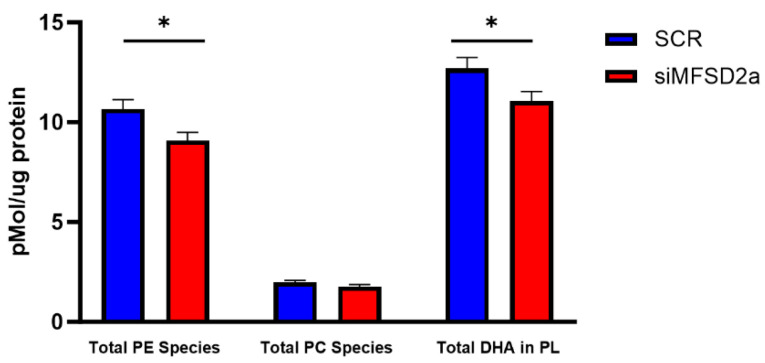
Total phosphatidylethanolamine (PE) DHA, phosphatidylcholine (PC) DHA and total (PC + PE) phospholipid DHA in fetal brain homogenates at E18.5 measured by LC-MS/MS in fetuses with placental shRNA-induced MFSD2a knockdown (*n* = 33) and SCR non-coding controls (*n* = 26). Two sample unpaired *t*-tests, * = *p* < 0.05.

**Figure 4:**
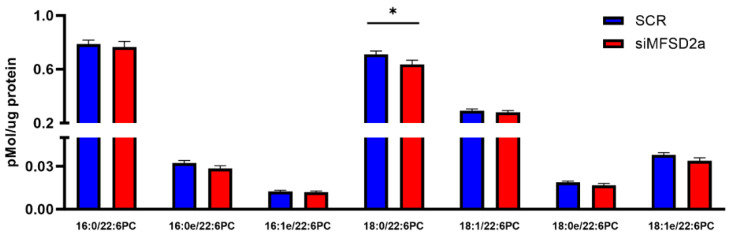
Phosphatidylcholine species containing DHA in fetal brain homogenates at E18.5 measured by LC-MS/MS in fetuses with placental shRNA-induced MFSD2a knockdown (*n* = 33) and SCR non-coding controls (*n* = 26). Two sample unpaired *t*-tests, * = *p* < 0.05.

**Figure 5:**
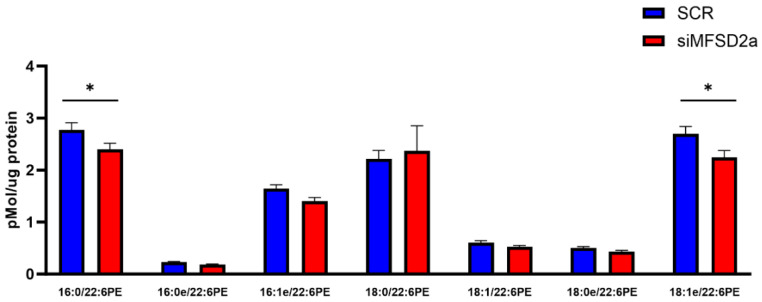
Phosphatidylethanolamine species containing DHA in fetal brain homogenates at E18.5 measured by LC-MS/MS in fetuses with placental shRNA-induced MFSD2a knockdown (*n* = 33) and SCR non-coding controls (*n* = 26). Two sample *t*-tests, * = *p* < 0.05.

**Figure 6:**
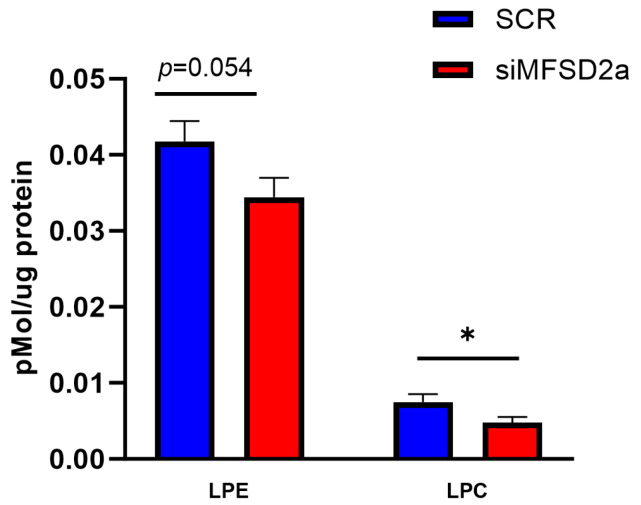
Lysophosphatidylethanolamine (LPE) species and lysophosphatidylcholine (LPC) species containing DHA in fetal brain homogenates at E18.5 measured by LC-MS/MS in fetuses with placental shRNA-induced MFSD2a knockdown (*n* = 33) and SCR non-coding controls (*n* = 26). Two sample *t*-tests, * = *p* < 0.05.

We analyzed fetal brain DHA content in two phospholipids, phosphatidyl choline and phosphatidyl ethanolamine, which make up 75% of brain phospholipids. Fetal brain was collected at E18.5 and the phospholipids were analyzed by LC-MS/MS in 33 brain samples from placental MFSD2a knockdown and 26 SCR controls. We found that in the fetuses with MFSD2a placental knockdown, total phospholipid DHA (PL-DHA) and phosphatidylethanolamine DHA (PE-DHA) were significantly lower per μg of brain protein (Figure 3). Total phosphatidylcholine DHA (PC-DHA) was not significantly reduced, but one of the major species of PC (18:0_22:6) was significantly lower in animals with placenta-specific MFSD2a knockdown (Figure 4). Likewise, several of the major PE species (Figure 5) were significantly reduced in animals with placental MFSD2a knockdown: PE (16:0_22:6); PE (18:0_22:6); and PE (18:1_22:6). The lysophospholipids containing DHA were also reduced in brain tissue with lysoPE DHA (*p* = 0.054), and lysoPC DHA (*p* < 0.05) lower in brains of pups with MFSD2a placental knockdown (Figure 6) We found no differences in fetal brain phosphotidyl inositol, phosphotidyl glycerol or phosphotidyl serine containing DHA or the corresponding lyso phospholipids for these groups. Therefore, brain growth was reduced, and highly abundant phospholipid DHA species in the fetal brain were lower when placental expression of the LPC transporter MFSD2a was experimentally reduced, despite the fact that brain expression of MFSD2a was not impacted. Brain PE and PC phospholipid levels in both groups can be found in Supplementary Table S2. We also evaluated placenta phospholipids containing DHA in 26 animals with MFSD2a knockdown and 22 SCR controls. Placental PE and PC containing DHA were not different in the two groups. See data in Supplementary Table S3.

We examined the relationships between placental MFSD2a mRNA expression and brain phospholipids containing DHA using Pearson’s correlation. We found several brain phospholipids containing DHA were significantly correlated with placental MFSD2a expression (Table 1). We found no correlations between brain weight and total PE + PC DHA content. 

## 4. Discussion

The important role for DHA in brain growth and development has led most obstetrical and pediatric medical societies to recommend supplementation with DHA during pregnancy and lactation [12] to ensure adequate supplies during the period of rapid brain DHA accumulation (third trimester to 2 years of age) [10]. Transplacental transport of DHA from the mother to the fetus is critical to ensure sufficient fetal supply of DHA, but the mechanisms of placental DHA transport are largely unknown. In this report we provide mechanistic data for a critical role for placental MFSD2a, a lysophospholipid transporter, for brain growth and DHA content in mice. We successfully reduced expression of MFSD2a specifically in the placenta by approximately 40% through transduction of the blastocyst with shRNA lentivirus and subsequent embryo transfer [19,21]. Importantly, MFSD2a expression levels in the fetal brain and other organs were not impacted. Placenta-specific gene targeting is a powerful tool allowing for mechanistic studies of placental function on fetal development without impacting fetal gene expression. Loss of MFSD2a only in the placenta resulted in a significant reduction in brain weight at term with no change in fetal, placenta or liver weights. In addition to being smaller, the brain content of several high-abundance DHA-containing phospholipids as well as lysophospholipid species were lower in animals with placenta-specific MFSD2a knockdown. These data are the strongest mechanistic evidence to date that placental MFSD2a transports lysophospholipids containing DHA to the fetal circulation and that lower placental expression of MFSD2a causes a reduction in fetal brain growth and DHA content. 

In 2014, Nguyen and coworkers reported that Major Facilitator Superfamily domain-containing 2a (MFSD2a) is expressed in the endothelial cells of the blood–brain barrier and provided evidence that it transports DHA in the form of lysophosphatidylcholine (LPC-DHA) [17]. More recently, transmembrane transport by MFSD2a has been characterized as a flippase mechanism [22,23]. It is energetically costly to transfer LPC-DHA due to the hydrophilic head group, and it is believed that this transfer is energized by the inwardly directed Na^+^ gradient rather than being directly linked to ATP hydrolysis. Recently, new structural data indicate that the Na^+^ binding site is near the phospholipid headgroup and may act to stabilize the molecule rather than be co-transported across the plasma membrane [23]. Mice deficient in MFSD2a have severe microcephaly, brain DHA deficiency, and learning and memory deficits [18]. Additional support for an important role for MFSD2a in normal brain growth and development include studies of human non-lethal inactivating mutations in MFSD2a, which result in microcephaly, due to reduced white matter volume, and intellectual disability [24]. MFSD2a has been shown to be critical for maintaining the integrity of the blood–brain barrier by regulating transcytosis and has been implicated in multiple neurological diseases including intracranial hemorrhage, Alzheimer’s disease, sepsis-associated encephalopathy, autosomal recessive primary microcephaly (MCPH) and intracranial tumors [25].

The concept of fetal DHA biomagnification was introduced by Crawford and co-workers who found DHA was higher as a percentage of total fatty acids in cord blood compared with maternal blood [26]. It is critical to acknowledge that non-esterified fatty acids (NEFAs) are transported down their concentration gradients and plasma non-esterified DHA concentration is consistently higher in maternal than in fetal plasma [27]. To achieve a higher percentage of DHA in total fetal fatty acid content compared with the mother, preferential transfer of DHA across the placenta is required. Several placental DHA transporters have been implicated in the uptake of DHA from the maternal circulation including Fatty Acid Binding Protein isoform 4 (FATP4) [28,29] and a plasma membrane placenta-specific DHA binding protein [30]. These transporters can mediate the uptake of DHA into the syncytiotrophoblast and likely contribute to biomagnification; however, the mechanisms for efflux of DHA from the syncytiotrophoblast across the basal plasma membrane to the fetal circulation remain understudied. Recently, Prieto-Sanchez and coworkers demonstrated that Major Facilitator Superfamily Domain containing 2a (MFSD2a), a lysophospholipid transporter, is expressed in the human placenta and that expression is lower in women with GDM [31]. Subsequently, we reported that MFSD2a is localized to the basal or fetal facing plasma membrane of the syncytiotrophoblast, the transporting epithelium of the human placenta [32], where it may contribute to transfer of DHA to the fetal circulation. 

An important factor recently recognized for DHA status in pregnancy is maternal choline status [33]. If preferential transport of DHA from the placenta to the fetus is dependent on synthesis of phospholipids, then choline levels must be adequate for incorporation of DHA into phosphatidylcholine followed by phospholipase A2 hydrolysis to generate robust intracellular levels of LPC-DHA available for transfer by MFSD2a. We have reported rapid synthesis of phospholipids in cultured primary trophoblast cells exposed to uniformly labeled ^13^C non-esterified fatty acids [34]. We incubated primary human trophoblasts with four uniformly labeled ^13^C-fatty acids, including ^13^C-DHA, in concentrations representative of maternal plasma at the end of pregnancy. After 24 h, approximately 40% of all phosphatidylcholine-DHA species contained ^13^C-labeled DHA. We found no evidence of de novo synthesis for phospholipids containing DHA; rather, the incorporation of DHA was primarily through the remodeling pathway. Remodeling of phospholipids is a known pathway that increases incorporation of LCPUFAs into plasma membrane phospholipids to ensure membrane fluidity and protein function, particularly in lipid rafts [35]. The phospholipid remodeling process also generates high levels of intermediate lysophospholipids, which in the case of trophoblasts, we propose are transported out of the cell by MFSD2a. 

We have previously demonstrated high levels of lysophosphatidylcholine DHA in human umbilical circulation [32], which could arise from transfer across the placenta or synthesis in the fetal liver. The current study was designed to specifically test the impact of reducing placental MFSD2a expression while brain and liver function remain unaffected. With approximately 40% lower placental MFSD2a expression, fetal brain weight and phospholipid DHA content were reduced, indicating that placental transfer of LPC-DHA is critical for accumulation of DHA and normal brain growth. While reducing placental MFSD2a expression impacted brain DHA phospholipid content, the impact was most evident in several high-abundance phosphatidylethanolamine-DHA (PE-DHA) species. We also found borderline reduced LPE-DHA and significantly reduced LPC-DHA in the brain in animals with lower MFSD2a placental expression. Whether the lower LPE levels are due to interconversion of PC and PE or MFSD2a acting as an LPE-DHA transporter is unclear. MFSD2a can transport lysophospholipids which have a zwitterionic headgroup and a hydrophobic hydrocarbon tail, including LPC, LPE and lysophosphatidylserine (LPS), but not lysophosphatidic acid [17,23]. Our previous report that MFSD2a is localized to the basal plasma membrane of the human syncytiotrophoblast supports that MFSD2a is important for the release of DHA in the form of lysophospholipids to the fetus, which ensures adequate accumulation of DHA in utero and supports fetal brain growth. 

MFSD2a was identified as a Na^+^-dependent lysophosphatidylcholine transporter in blood–brain barrier endothelial cells [17,36]. The inwardly directed Na^+^ gradient can facilitate the uptake of nutrients through a secondary active transport mechanism. However, release of LPC-DHA through MFSD2a in the basal membrane of the placenta would not be assisted by Na^+^ co-transport. Recent functional models based on the MFSD2a structure suggest a flippase action for transferring LPC-DHA from the outer to the inner leaflet of the plasma membrane and suggested that Na^+^ binding is needed to stabilize the phosphocholine headgroup but that Na^+^ transport may not occur [23]. How lysophospholipids are released from the blood–brain barrier endothelium and taken up by astrocytes and neurons remains unclear [37]. In order to re-esterify lysophospholipids to phospholipids, acyltransferase enzymes located in the endoplasmic reticulum and mitochondria are required [35]. At the placental barrier, localization of MFSD2a to the basal membrane would allow LPC-DHA to be taken up by fetoplacental endothelial cells or to enter the capillaries through known endothelial intercellular gaps and bind to fetal albumin for transfer to the fetal brain. 

MFSD2a has been recognized as the receptor for Syncytin 2, a fusion-promoting protein expressed at high levels in the placenta. Fusion of mononucleated cytotrophoblast cells is critical for formation of the syncytiotrophoblast epithelium of the placenta. Localization of MFSD2a to the basal plasma membrane surface of the syncytium [32] would promote fusion of cytotrophoblasts localized below the epithelium. This fusion event preserves the microvillous or apical surface while allowing for expansion of the surface area of the placental transporting epithelium throughout gestation. A potential role for MFSD2a as a DHA transporter in the placenta has been suggested in pregnancies complicated by GDM and preeclampsia in which maternal DHA and cord blood DHA are not well correlated. In GDM pregnancies, higher maternal DHA has been reported, but these pregnancies are also complicated by lower DHA in the fetus [38]. In preeclamptic pregnancies, maternal, placental and cord blood DHA levels are all reduced [39,40], as well as expression of MFSD2a [41]. These pregnancy complications are also associated with neurological disorders in children [42,43]. Additional evidence for MFSD2a functioning as a DHA transporter in the placenta includes (1) high levels of LPC-DHA in the placenta, (2) a correlation between expression of the MFSD2a transporter in the fetal-facing basal plasma membrane and cord blood LPC-DHA, and (3) high umbilical cord levels of LPC-DHA [31,32]. Therefore, in addition to a role in cytotrophoblast fusion and placental growth, MFSD2a may be essential for release of LPC-DHA to the fetal circulation. Our current study in mice supports a role for placental MFSD2a in fetal brain growth and DHA content, again suggesting that lysophospholipid-DHA is released from the syncytiotrophoblast to the fetus by MFSD2a. Localization studies for MFSD2a in the blood–brain barrier and human placental endothelial cells are needed to determine whether MFSD2a is acting as an influx or efflux transporter, or both, depending on the concentration gradient of LPC-DHA inside and outside the cell. 

## 5. Conclusions

Our study suggests that placental MFSD2a expression may be an important factor determining how effectively maternal DHA reaches the fetal brain. Surprisingly, regulation of the expression of MFSD2a has not been studied in detail in any tissue [44]. This information will be critical for improving MFSD2a expression and function in pregnancies in which maternal DHA status is low and supplementation is needed to improve fetal brain uptake of this vital nutrient. While the implications for long-term neurological health of the offspring were not tested in our study, previous studies in experimental animals and in humans have demonstrated the detrimental effects of perinatal DHA deficiency on learning, memory, cognitive function and neurological disorders [10].

## Figures and Tables

**Table 1:** Pearson correlation coefficients between brain phospholipid levels and RQ MFSD2a.

Brain Phospholipid	Correlation Coefficient	*p* Value
PE (16:0_22:6)	0.28	**0.0317**
PE (16:0e_22:6)	0.30	**0.0219**
PE (16:1e_22:6)	0.26	**0.0429**
PE (18:0_22:6)	0.27	**0.0409**
PE (18:1_22:6)	0.22	0.0943
PE (18:0e_22:6)	0.28	**0.0324**
PE (18:1e_22:6)	0.28	**0.0288**
LPE (22:6)	0.21	0.109
Total PE containing DHA	0.32	**0.0122**
PC (16:0_22:6)	0.17	0.211
PC (16:0e_22:6)	0.22	0.0919
PC (16:1e_22:6)	0.13	0.3236
PC (18:0_22:6)	0.26	**0.0474**
PC (18:1_22:6)	0.16	0.2302
PC (18:0e_22:6)	0.25	0.0527
PC (18:1e_22:6)	0.25	0.0546
LPC (22:6)	0.19	0.1533
Total PC containing DHA	0.21	0.1114
Total Brain PE + PC DHA	0.31	**0.0166**

Bold text indicates significant correlations between lipid species listed and placenta mRNA expression for MFSD2a.

## Data Availability

Data are contained within the article and supplementary materials.

## Supplementary Materials

The following supporting information can be downloaded at: https://www.mdpi.com/article/10.3390/nu15234956/s1, Table S1. Primers for QPCR. Table S2. Phospholipid levels in fetal brain at E18.5. Table S3. Phospholipid levels in placenta at E18.5. Figure S1: Fetus at E18.5 with placental MFSD2a knockdown (A) under a UV lamp to detect expression of EGFP and under (B) normal light. Placentas show fluorescences at the visible green light with the peak of GFP at wavelength 509 nm. The fetus shows some skin autofluorescence and appears a yellowish color. Autofluorescence (visible in yellow) peaks around 595 nm, our filter was not specific enough to only detect the green visible light.
