# Supplementary material for: Knockdown of Placental Major Facilitator Superfamily Domain Containing 2a in Pregnant Mice Reduces Fetal Brain Growth and Phospholipid Docosahexaenoic Acid Content
Source: Nutrients. 2023 Nov 29;15(23):4956. doi: 10.3390/nu15234956 (PMC10708493; doi:10.3390/nu15234956)
Supplement: Supplementary file 1 [file nutrients-15-04956-s001.zip › Tables S1-S3.pdf]

Supplemental Tables

Supplementary Table S1. Primers for QPCR.

**Target mRNA**	**Forward primer**	**Reverse Primer**
Mouse Mfsd2a	AGAAGCAGCAACTGTCCATTT	CTCGGCCCACAAAAAGGATAAT
Rpl19	ATGAGTATGCTCAGGCTACAGA	GCATTGGCGATTTCATTGGTC

Supplemental Table S2. Phospholipid levels in fetal brain at E18.5.

Phospholipid	MFSD2a (n=33)	SCR (n=26)	P Value
PE (16:0_22:6)	2.4±0.68	2.77±0.72	**0.0492**
PE (16:0e_22:6)	0.19±0.06	0.23±0.07	**0.0183**
PE (16:1e_22:6)	1.4±0.4	1.64±0.37	**0.0208**
PE (18:0_22:6)	1.89±0.57	2.22±0.8	0.087
PE (18:1_22:6)	0.52±0.15	0.6±0.19	0.0862
PE (18:0e_22:6)	0.43±0.15	0.5±0.14	0.0612
PE (18:1e_22:6)	2.25±0.75	2.7±0.71	**0.0218**
LPE (22:6)	0.03±0.01	0.04±0.01	0.0517
Total PE containing DHA	9.08±2.4	10.66±2.38	**0.0144**
PC (16:0_22:6)	0.77±0.24	0.85±0.23	0.2030
PC (16:0e_22:6)	0.03±0.01	0.03±0.01	0.0731
PC (16:1e_22:6)	0.01±0.01	0.01±0	0.3503
PC (18:0_22:6)	0.64±0.18	0.73±0.17	**0.0423**
PC (18:1_22:6)	0.28±0.08	0.3±0.07	0.2301
PC (18:0e_22:6)	0.02±0.01	0.02±0.01	0.2233
PC (18:1e_22:6)	0.03±0.01	0.04±0.01	0.1183
LPC (22:6)	0±0	0.01±0.01	0.0421
Total PC containing DHA	1.77±0.52	1.99±0.48	0.1079
Total Brain PE+PC DHA	10.89±2.89	12.7±2.8	**0.0184**

Supplemental Table S3. Phospholipid levels in placenta at E18.5.

Phospholipid	MFSD2a (n=26)	SCR (n=22)	P Value
PE (16:0_22:6)	0.41±0.17	0.42±0.17	0.8502
PE(16:0e_22:6)	0.06±0.02	0.05±0.02	0.7421
PE (16:1e_22:6)	0.72±0.29	0.71±0.3	0.8456
PE (18:0_22:6)	1.12±0.36	1.11±0.35	0.9257
PE (18:1_22:6)	0.13±0.07	0.12±0.07	0.5541
PE (18:0e_22:6)	0.09±0.03	0.09±0.04	0.931
PE (18:1e_22:6)	0.96±0.37	0.97±0.42	0.9208
LPE (22:6)	0.09±0.04	0.09±0.04	0.9992
Total PE containing DHA	3.583±0.25	3.563±0.29	0.9574
PC (16:0_22:6)	0.72±0.36	0.76±0.37	0.733
PC (16:0e_22:6)	0.13±0.07	0.14±0.07	0.7208
PC (16:1e_22:6)	0.13±0.08	0.13±0.07	0.8807
PC (18:0_22:6)	1.29±0.6	1.35±0.62	0.7486
PC (18:1_22:6)	0.09±0.06	0.09±0.05	0.9288
PC (18:0e_22:6)	0.13±0.07	0.15±0.1	0.4936
PC (18:1e_22:6)	0.16±0.09	0.16±0.09	0.7937
LPC (22:6)	0.1±0.04	0.11±0.04	0.75
Total PC containing DHA	2.66±1.29	2.78±1.34	0.7539
Total Placenta PE+PC DHA	6.34±2.58	6.45±0.57	0.8925
