# Supplementary figures and images for: Knockdown of Placental Major Facilitator Superfamily Domain Containing 2a in Pregnant Mice Reduces Fetal Brain Growth and Phospholipid Docosahexaenoic Acid Content
Source: Nutrients. 2023 Nov 29;15(23):4956. doi: 10.3390/nu15234956 (PMC10708493; doi:10.3390/nu15234956)

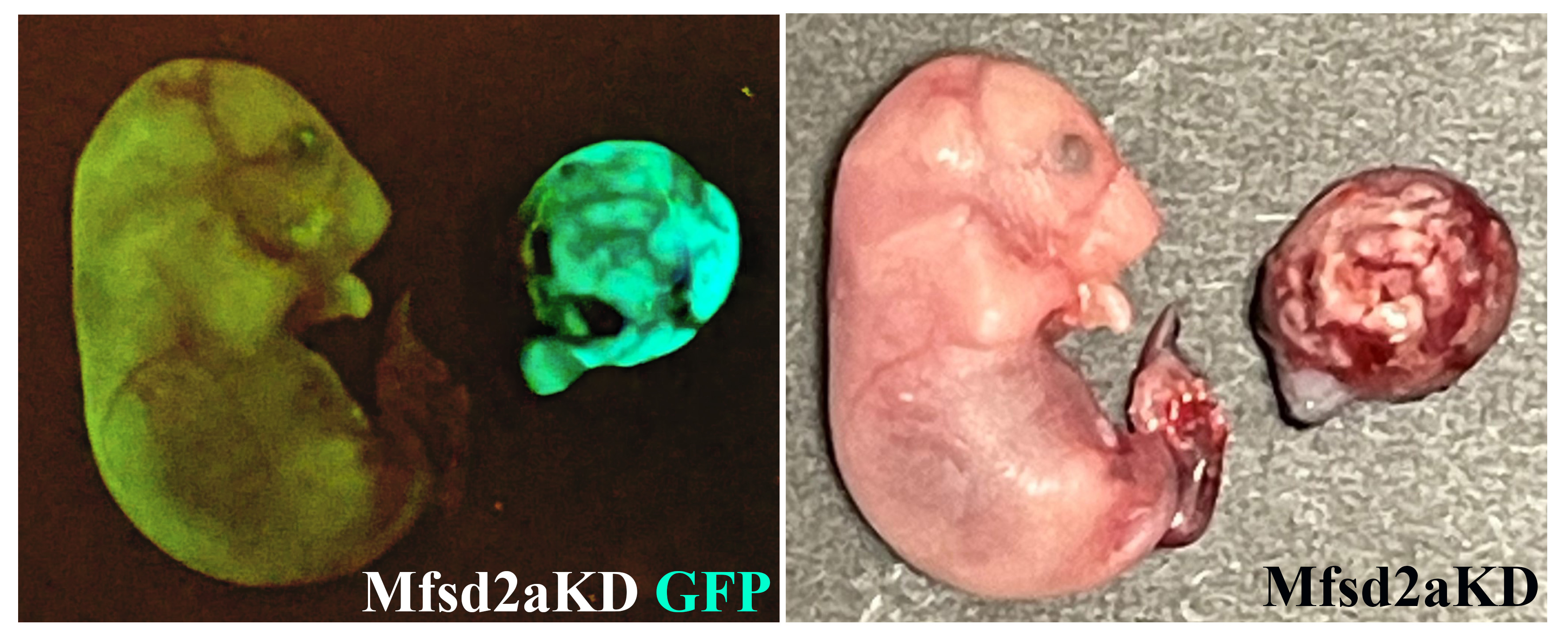

Supplement: Supplementary file 1 [file nutrients-15-04956-s001.zip › Figure S1.jpg]
